# Supplementary material for: General practitioner perspectives on factors that influence implementation of secondary care-initiated treatment in primary care: Exploring implementation beyond the context of a clinical trial
Source: PLoS One. 2022 Oct 17;17(10):e0275668. doi: 10.1371/journal.pone.0275668 (PMC9576064; doi:10.1371/journal.pone.0275668)
Supplement: S1 Data — (DOC) [file pone.0275668.s001.doc]

GP Interview Schedule: BOPPP Trial

*Telephone interviews to investigate how beta-blockers for primary prophylaxis of oesophageal varices can be best delivered in primary care*

*The aim of this interview is to explore GP perceptions regarding the use of beta-blocker treatment for patients with small oesophageal varices in routine primary care practice. Participants will be encouraged to discuss their opinions, experiences and perceptions in an open way to ensure any issues of importance to the BOPPP trial are not excluded and a diversity of responses are gathered. The information gathered will be used to support future implementation and to improve uptake of this treatment summary beyond the trial context.*

*The interview schedule contains key questions and themes to be explored. Further questioning will fully explore participants’ contributions in order to understand how and why views are held.*

Participant information sheet provided in advance. Participants given the opportunity to ask questions, and sign consent form.

1. Introduction to interview and ground rules

*Aim: to introduce the research and set the context for the interview*

- **Introduce interviewer**
- **Introduce the interview study**

The purpose of the study is to find out about your views and experiences of implementing the BOPPP trial treatment strategy as a part of routine clinical care. This provides us with information on factors that facilitate or hinder implementation and on possible solutions to overcome any identified barriers.

*We are conducting a trial to compare carvediolol and placebo in the prevention of variceal bleeding for patients with small oesophageal varices. We would like to invite GPs to take part in a telephone interview as part of this research because we are interested in experiences regarding the use of beta-blocker treatment for patients with small oesophageal varices in primary care practice.*

- **Details about participation**

Voluntary nature of participation – both overall and in relation to any specific questions and discussions

- - Length of interview - around 1 hour. Will finish on time – clock to keep to time
  - Recording of interview – stop for loud noises
  - Confidentiality and how findings will be reported - anonymity, transcribing
  - Emphasise there is no need to share personal experiences unless people want to
  - Explain that there are no right or wrong answers – interested in a range of views,

opinions and experiences

- **Basic ground rules**
  - Mobile phones off (or on silent/vibrate)
- Any questions?

1. Background

*Aim: to introduce context of the BOPPP trial.*

- To start, we will provide a brief overview of the BOPPP trial and the context of beta-blocker use as defined by this study. NB: Remain mindful that this context may be further away that what is currently happening in services.

1. Introductions for participants – **Start recording session** (5 mins)

- Our overall aim is to promote the use of beta-blocker treatment for patients with small oesophageal varices in routine primary care practice.

*We would like to ask you about what helps and what hinders success in implementing the beta-blocker treatment strategy as a part of routine clinical care.*

1. Understanding implementation of beta-blocker use

*Aims: 1) To explore what participants think helps or hinders implementation.*

*2) To explore what participants identify as potential solutions to implementation barriers.*

3a. **What helps implementation?** Facilitating factors (25 mins)

- **What factors do you perceive might support implementing the BOPPP treatment strategy in primary care?**
- **What would help you to provide beta-blocker treatment as a part of routine clinical care?**
- **What are your views on the BOPPP trial and acceptability of the BOPPP treatment strategy?**
- **Are there/or might there be challenges around dose titration for patients with small oesophageal varices within primary care?**

*Probe:*

- **What can you tell us about that?**
- **Are there any other factors that hinder?**
- Bring it back on topic… **Can I ask a question related to that, that I asked earlier – what hinders you and your team to promote beta-blocker use in this context?**
- Uncertainty of participant in answering question… **Which way is most important to you?**

3b. **What prevents or hinders implementation?** Barriers (25 mins)

- **What prevents or hinders success in implementing the beta-blocker treatment strategy as a part of routine clinical care?**

*If implementation has been perceived as low:*

1. **What would further support you to implement the BOPPP treatment strategy?**

Prompts / Continuation Questions:

- What resources would better equip you to implement the BOPPP treatment strategy?
- What would you say is the ideal environment to implement the BOPPP treatment strategy in?

*Probe:*

- **What can you tell us about that?**
- **Are there any other factors that hinder?**
- Bring it back on topic… **Can I ask a question related to that, that I asked earlier – what hinders you and your team to promote beta-blocker use in this context?**
- Uncertainty of participant in answering question… **Which way is most important to you?**

3c. Implementation solutions (25 mins)

- **What solutions would you recommend to address these identified barriers?**
- **Are there any strategies and potential solutions to early dose adjustment?**
- **Any other influences on implementation? COVID? Other clinical priorities?**
- **Are there any other important points that you would like to discuss before we close the discussion?**

Bring discussion to a close, thank respondent and reiterate confidential nature of interview. Any further questions about us or the research?

Explain what happens next – involvement in RCT and when they might next hear from BOPPP.
