# Supplementary material for: General practitioner perspectives on factors that influence implementation of secondary care-initiated treatment in primary care: Exploring implementation beyond the context of a clinical trial
Source: PLoS One. 2022 Oct 17;17(10):e0275668. doi: 10.1371/journal.pone.0275668 (PMC9576064; doi:10.1371/journal.pone.0275668)
Supplement: S2 Data — (DOCX) [file pone.0275668.s002.docx]

Full coding framework

**Theme 1: Primary care is well-placed for oversight**

- 1. **Preventative medicine is core work**
     1. Acknowledging the risks of untreated varices
     2. First-hand experience of varices
     3. Improving patient lives
     4. Reducing costs for health care
  2. **Fits with holistic and patient centred practice**
     1. Balance risks and benefits and take account of other health conditions, context and environment
  3. **Existing relationships with patients**
     1. The GP practice is a constant
  4. **Established pathways for medication reviews**
     1. Starting new medication is something we do all the time
     2. Just another part of the medication review
     3. It becomes routine as time goes on
  5. **Existing practice-based approach**
     1. Draw on the skills of the PCN team
     2. Changing role of the GP
  6. **Treatment will have an evidence base**
     1. Acceptability of treatment and monitoring regime
        1. Familiarity of Carvedilol
        2. Well-known and safe drug
        3. Used comfortably in other situations

**Theme 2: A shared approach led by secondary care**

- 1. **Joined up thinking and working together**
     1. The concept of shared care
     2. Start the conversation in secondary care
     3. A chance for patients to ask the specialist questions
     4. Stabilise the dose in secondary care
     5. Provides time for communication and organising medication supply (e.g., 14 days)
     6. Identify who is responsible clinician
  2. **Ongoing open communication between secondary and primary care**
     1. A point of contact and access to specialist advice
     2. Email is most efficient
  3. **A clear management plan from secondary care**
     1. Include what to do and how to do it
     2. Individualise dose titration/monitoring needs for each patient
     3. Integrate into patients notes
  4. **Integrate into primary care systems**
     1. Set up electronic system reminders
        1. Future alerts (i.e., one year on)
     2. Available on the shared drive for commissioners

**Theme 3: Empower the patient to take responsibility**

- 1. **Time and resources to engage patients**
     1. Specific needs of the patient group
     2. Social factors
     3. Working with other services such as drug and alcohol and mental health services
     4. Supporting patients when asymptomatic
  2. **Patient education**
     1. Patient information leaflet
     2. Letter advising them to contact GP
  3. **Shared decision making**
     1. Conversations with patients to discuss the pros and cons
        1. GP information sheet to guide conversation
     2. Develop decision aids
        1. Different forms and varied information
        2. Involve patients and healthcare practitioners in development

**Theme 4: The need to go above and beyond**

- 1. **Adding to the workload**
     1. 100 plates spinning
  2. **Finding a way to prioritise it**
     1. Different drugs and treatments are fighting to be heard
  3. **It is not a common problem so how relevant is it to me**
     1. Better systems for the things we see frequently
     2. Infrequency means it takes more time because it is not routine practice
     3. Unfamiliarity, limited experience, and knowledge
        1. A cautious approach and low confidence
  4. **Taking it on in covid times**
     1. Remote monitoring
     2. Rapid decision-making
  5. **Consider the need for financial reimbursement**
     1. Claims can be more laborious than the income

**Theme 5: Develop practice guidance**

- 1. **Involve GPs in implementation conversations at the outset**
  2. **Report the evidence**
     1. Include in NICE/British Society for Gastroenterology/Hepatology guidance
  3. **Develop implementation guidelines**
     1. Develop a medication pathway for commissioning
     2. Link with medicines group CCG
  4. **Optional training**
     1. Quick, easy, accessible and at our fingertips
     2. Hot topic updates, Webinar, learning day
